# Spatial and Social Sexual Segregation Patterns in Indo-Pacific Bottlenose Dolphins (*Tursiops aduncus*)

**DOI:** 10.1371/journal.pone.0052987

**Published:** 2013-01-09

**Authors:** Christine Ann Fury, Kathreen E. Ruckstuhl, Peter L. Harrison

**Affiliations:** 1 Southern Cross University Marine Ecology Research Centre, Southern Cross University, Lismore, New South Wales, Australia; 2 Department of Biological Sciences, University of Calgary, Calgary, Alberta, Canada; University of Western Ontario, Canada

## Abstract

Sexual segregation seems to be common in bottlenose dolphins, whereby males and females live in different pods that mix mainly for mating. Male dolphins often use aggressive behaviour to mate with females, while females with calves may have different activity and dietary requirements to males and different susceptibility to predation. We investigated the degree of spatial and social sexual segregation in Indo-Pacific bottlenose dolphins (*Tursiops aduncus*) in a subtropical estuary in Australia. Based on surveys completed over three years, dolphin groups were mostly mixed-sex or female. Mixed-sex groups were found in larger groups in mostly deeper water, whereas, female groups were foraging across all water depths in smaller groups. Aggressive coercive behaviour by males towards females was high, occurring mainly in deeper water, at higher tides, and outside the breeding season. Habitat use by female dolphin groups suggests that shallow tributaries may provide a sanctuary from aggressive males, access to suitable prey items and density for mothers and their calves, or a combination of these factors.

## Introduction

Sexual segregation for much of the year is a widespread phenomenon among social mammals including, ungulates [1], bears [2], kangaroos [3], seals [4], [5] and cetaceans [6]–[9]. Sexual segregation theory divides the categories of sexual segregation into social segregation where sexes live in separate groups outside of the breeding season, habitat segregation where sexes differ in habitat use, and spatial segregation where they occupy different areas within the same habitat [10]. A number of different hypotheses have been proposed to explain sexual segregation including the activity budget, predation risk, forage selection and male-avoidance strategy hypotheses [1], [11]–[13].

Little is known about the degree of spatial or sexual segregation in bottlenose dolphins or factors that could be driving these patterns. Our study thus aimed at quantifying the types of dolphin groups most commonly found throughout the different seasons, what types of habitat they use, and, if spatial or social sexual segregation occurs, which variables might explain these patterns. The main predators of bottlenose dolphins in the study site are bull sharks (*Carcharhinus leucas*) that are found mainly in the deeper estuary channels during the day; an area that dolphins and particularly females with young calves should avoid [14]. It has also been proposed that females with calves might avoid male harassment [15] and thus avoid areas frequented by males. It has further been proposed that females might avoid males due to direct or indirect effects on the frequencies of antagonistic interactions: females might avoid males of all age classes either because males engage in agonistic acts in the presence of females [11] or because male presence causes an increase in female-female aggression as has been shown for Roosevelt elk [12], [13].

The social ecology of bottlenose dolphins is quite variable geographically, however, it has been described through several long-term studies [16]–[18] to show a complex fission-fusion society whereby dolphins join and leave groups on a flexible basis, with interactions between individuals lasting for minutes to years. Sexual segregation is thought to be the basic framework of the society for some *Tursiops* spp. For dolphins that show sexual segregation it is strongest in the adults, where females with calves are a key element forming female groups, groups of males form strongly bonded alliances among 2–3 adult males, and juveniles form groups of mixed sexes [19]–[22]. Alliances are cooperating male dolphins working in pairs and triplets to seize and control the movements of females [16], [23]. These alliance formations have been recorded in Port Stephens and Shark Bay, Australia and the Bahamas [19], [24]–[26]. However, other studies also show that male alliances are not the predominant feature in all bottlenose dolphin populations and the degree of sexual segregation and the proportion of mixed-sex groups vary considerable geographically. For instance, common bottlenose dolphins (*T. truncatus*), such as those in Europe show no strong alliances between males and often dolphins occur in mixed-sex groups [27], [28].

The aim of this study was to describe the social system, seasonal and gender differences in habitat use and social segregation, and to investigate to what extent sexual segregation (spatial, habitat or social) occurs in a population of Indo-Pacific bottlenose dolphins (*T. aduncus*) in a subtropical estuary in eastern Australia. We predicted that female dolphins use different habitats than males, partly to avoid male aggression. Also, as small fish are more abundant in shallow estuarine waters, which makes it easier for calves to catch and consume small fish, we expected females with calves to be found in shallower waters compared to males. It was also expected that the dolphins would use different habitats during different tides and seasons according to prey availability, and that this would be independent of sex. It has been shown that differences in activity budgets between the sexes can lead to social segregation [29]. Furthermore, because of high energy demands due to lactation, we expected lactating females to show different activity budgets and specifically to spend more time foraging than adult males [30].

## Materials and Methods

### Study Site and Population

**Figure 1:**
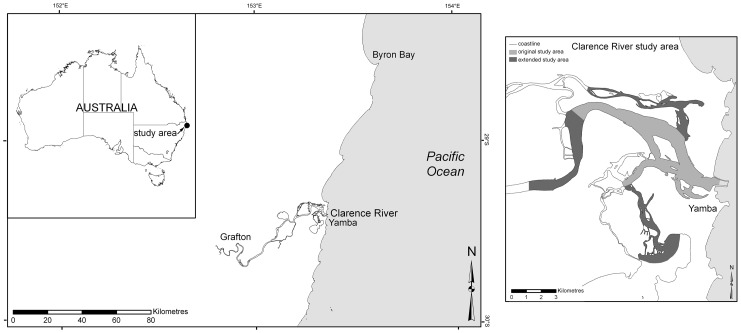
Map of the study sites in northern New South Wales, Australia at the top and a detailed map of the Clarence River estuaries indicating the survey areas.

The Clarence River estuary (CR) is located on the subtropical north coast of New South Wales (NSW) (Figure 1). It is the largest coastal river in northern NSW [31] with a catchment area of approximately 22,446 km^2^, a waterway area of 89 km^2^, volume of 204.7×10^6^ m^3^ and a mean depth of 2.3 m. The approximate length of the river tidal influence is about 60 km from the mouth. The mean spring tidal range at the entrance is 1.34 m [32], [33].

### Dolphin population

Results from the broader study on this dolphin population [34] concluded that the CR sustains a moderately sized, predominantly resident dolphin community. The abundance estimate from mark-recapture analyses were 71 (62–81 95% CI, CV = 0.07) dolphins utilizing the CR [34]. Site fidelity patterns of identified dolphins determined that 60% were residents, 26% were occasional visitors, and 14% were transients [34]. The dolphins were found to utilise the estuary all year-round and their spatial distribution was largest on high and flood tides [35].

### Field Methods

Boat-based surveys were completed in the Clarence River (CR) estuary over 3 years from October 2003 to September 2006 (4 to 12 surveys per calendar season). Surveys were conducted using small aluminium boats (∼4 m) powered by a 2-stroke engine. During the surveys the boat was kept at a steady speed of ∼6 knots and 1–3 observers maintained a constant visual search for dolphins. All surveys were done in calm conditions (i.e., Beaufort sea state ≤3) between 06:00 and 18:00 h. Generally, surveys were conducted to include all daylight hours, with morning surveys occurring from 7:00–13:00 and on other days afternoon surveys at 11:00–17:00 in most seasons, and with surveys starting earlier and finishing later when longer periods of daylight were available, particularly during summer. Different times of the day, tidal periods, weather conditions, months and all seasons were used for surveys to encompass all possible ranges and to avoid bias from only observing animals in the mornings or afternoons and potentially missing important behaviours that might only be displayed at certain other times of the day, only during ebb or flood tides, or only during certain months. The route for the surveys was randomized to ensure that surveys started and finished at different locations each survey, and travel occurred along different channels until the whole study area was covered. If dolphins were not located, surveys were still continued for the full survey time period to encompass multiple surveys of the full study area. Surveys ranged over a 16 km^2^ area, which included surveys up to ∼28 km upstream from the river mouth. The study area began from 500 m landward of the estuary mouth. From 2005, if the dolphins were found in the study area and travelled upstream past the end of the designated study area they were followed up to a further 24 km. The surveys used a zigzag pattern in the wider areas of the estuary to maximize the chances of any encountering dolphins present, and the entire survey area was always covered during each survey period. A group of dolphins was defined as individuals being within a 100 m radius of one another and engaged in similar activities [36].

#### Group composition

65% of adult dolphins were individually identified by marks on their dorsal fins [34]. The group's gender classification was determined as males, females, mixed-sex, or unknown. Groups were classified as a mixed-sex group if at least one adult male and one adult female were present. Groups were considered female only groups if all adults, not juveniles, were known females. For female groups, a dolphin was classified as female by the consistent presence of a calf, sighted at least 6 times together, at any time in the 3 years of the study, or by observation of the genital slit. Males were classified by direct observation of their penis. Most often sex identification would involve post-fieldwork analysis of photographs where an erect penis was observed. If a gender category could not be established for the group, i.e. all adults could not be identified, it was classified as unknown (29%) and the data were not used in the analyses.

Focal group follows were conducted during dolphin surveys where a focal group was followed to whatever extent possible during each sample period. Groups were followed from a distance of between 1–50 m, however, mostly at 30 m as prescribed by permit limits; however, sometimes dolphins came closer to the survey boat. An encounter with a group occurred until i) dolphins were no longer able to be observed, ii) the group split up or joined another group, or iii) the 3 hour limit had been reached as directed by the ethics permit. The survey then continued by searching for another group of dolphins. Instantaneous scan sampling involved an observer recording the group's current behaviour at pre-selected moments in time [37]. For this study, behaviour was recorded every 3-mins on the minute throughout the observation period to determine the activity budget of the dolphins.

A total of 87 boat-based surveys were completed during the 3-year study comprising of 468.5 h, with 142 dolphin groups and 2,452 dolphin GPS habitat use points for the full data set and 89 GPS habitat use points for the subset (only one data point per encounter). The median survey duration was 360 mins (range 120–510 mins). Lengths of focal follows of dolphins were between 12–180 mins. Survey effort (X^2^_3_ = 1.27, *P* = 0.74) and direct dolphin observation time (X^2^_3_ = 1.2, *P* = 0.753) were not significantly different among seasons (Table 1).

**Table 1:** Boat-survey effort and direct dolphin observation (DDO) periods during each season.

*Study Sites*	*Clarence River estuary*	
*Season (year)*	*Survey effort (h)*	*DDO (h)*
Spring (2003–5)	112	35.5
Summer (2003–5)	122	26.7
Autumn (2004–6)	124	29
Winter (2004–6)	111	28.7
Total (3 years)	469	120

#### Spatial patterns and Activity Budget

Upon sighting a group of dolphins, the group was slowly approached to minimize disturbance and the group position was determined using a hand-held global positioning system (GPS) (Garmin GPS 12), and the behavioural states were recorded. Behavioural states were foraging, travelling, social interactions and rest/milling. Social interactions were further categorized by the presence or absence of coercive behaviour by males if that was observed. Coercion is a form of aggressive behaviour used by males that occurs frequently among animals with promiscuous mating systems and often involves males using force to increase the chances that a female will mate with male/s [38], [39]. In bottlenose dolphins, Connor *et al.* [25] observed that coercion often involves aggressive behaviour by males towards a female including biting, hitting with the tail, head-jerks or body slamming the female. The aggressive coercive behaviour observed in the CR was defined as 3–4 male dolphins flanking a female dolphin, often with all three males having erect penises and trying to force copulation with the female. There were often calves or juveniles accompanying the group, however, they were not herded by the males but remained within 10 m of the group.

The GPS data points were downloaded using Waypoint + [40] and converted into GIS format using the program ArcView (ESRI, v.9.0). The group composition data were combined with the photographic identification data and sorted by groups, not individuals, and group composition by gender was determined for the adults in the group (not calves). A minimum of four GPS behavioural data points was required for the group to be considered in the analyses [41], however, to avoid auto-correlation associated with the 3-min sampling method with differing encounter lengths, only one data point per encounter was used for the analyses of behaviour. The data point used was that recorded at the 12^th^ minute mark (i.e. 4^th^ data point); this data point was used because by this time any slight change in behaviour that might have occurred due to the presence of the boat had ceased [42].

All data on groups of known composition were then analysed for each of the dolphin gender group categories (male, female or mixed-sex) for habitat utilization patterns, using the kernel density estimator (KDE) analysis with least squares cross validation [43], [44] in ArcView (ESRI, version 3.2) and Animal Movement Analyst Extension (AMAE) [44]. The fixed kernel density estimator is regarded as the most appropriate method for estimating habitat utilization distribution (see [45]–[47]), and uses probability density functions to identify areas of intense use [43]. Many other methods have been developed since then, but the MCP and the kernel remain the most widely used and comparable approaches (see reviews in [48]–[50]). We used least-squares cross-validation (LSCV) as it is the most commonly used method for automatically calculating the smoothing parameter [45], [46]. The LSCV method attempts to find a value for h that minimizes the mean integrated square error (MISE) by minimizing a score function CV(h) for the estimated error between the true density function and the kernel density estimate [51]. We used the fixed kernel approach that assumes the width of the standard bivariate normal kernel placed at each observation is the same throughout the plane of the utilization distribution.The 30% and 50% isopleths were used to investigate the core areas utilized by the dolphins, and the 75% isopleth shows the broader use of these areas [45], [52], [53].

#### Sexual differences in activity budget, spatial or habitat use

The dependant variable was gender groups (male, female and mixed-sex). Each dolphin gender group encounter was classified into these predictor variables; duration of encounter (mins), year of survey (1, 2 or 3), time of day (1, 2, 3, and 4), behaviour (foraging, travelling,social interactions, and rest/milling), tidal phase (high, low, flood, ebb), tidal range (spring, neap), water depth (deeper, medium or shallow), group size and season (spring, summer, autumn, winter). Categories used for the variables were as follows. Year of survey categories were; Year 1 = spring 2003–winter 2004, Year 2 = spring 2004–winter 2005, Year 3 = spring 2005–winter 2006. Time of day categories were; 1 = 06:00–09:00 h, 2 = 09:00–12:00 h, 3 = 12:00–15:00 h, 4 = 15:00–18:00 h.

The deeper water category occurred in the main channel where the average depth was 6–12 m, medium water depth was 4–5 m average depth with some sand banks present, and shallow water depth was 1–3 m average depth with many sand banks scattered throughout the area. Depth data and habitat type were determined from geomorphic habitat maps [42], [54] and from records of water depth obtained using a Secchi disk as a weighted line to measure water depth at each survey location for associated research at these sites [42], [55]. Seasons were spring (September–November), summer (December–February), autumn (March–May) and winter (June–August).

Tidal phase was divided into three-hour periods with high tide including the hour of high tide and the hour prior to and subsequent to it. Low tide included the hour of low tide and the hour prior to and subsequent. Flood tide included the 3 hours between the low tide and high tide periods, and ebb tide included the 3 hours between high tide and low tide periods. Tidal ranges included spring tides that were around the full moon and new moon period, and neap tides that were on the waning and waxing moon period and were of equal duration between categories.

#### Statistical Analysis

All of the predictor and dependent variables are categorical or nominal in nature. We therefore mostly used Chi-square analyses. Analyses were performed using the software package JMP [56] and SPSS for windows [57]. Analyses were performed using either the full data set or a subset. In the subset data set we only used one data point per encounter with a particular group on a given day to avoid problems of pseudoreplication. To test for sexual differences in activity budgets we performed Chi-square tests on the full data set, with behavioural frequencies as the dependent and gender group categories as the predictor variable. To test for seasonal differences in the frequencies of female and mixed-sex groups we used a Mann-Whitney U-test on the subset data. To test whether the sexes use different habitats we compared seasonal habitat use for each gender group category using a Chi-square test with water depth category as the dependent and gender group category as the predictor variables. We also tested if the dolphins were using the different water depths (dependent variable) differently according to season and gender groups (predictor variables). Male-only groups were not included in any of the analyses because of insufficient data available for these groups. A Kruskal-Wallis test was used on the full dataset, separately, for each gender group to determine independence. We also wanted to identify environmental factors that could contribute to sexual segregation patterns, and thus tested for the effect of year of survey, time of day, behaviour, group size, duration of encounter, tidal phase, tidal range, water depth and season (predictor variables) on the occurrence of mixed-sex or female-only groups, using a nominal logistic model, with group type as the dependent variable.

We also investigated whether different frequencies of coercive behaviour (dependent variable) were associated with different water depths, tides, and seasons (predictor variables) using Chi-square tests.

## Results

### Gender group categories, Activity budgets and Group size

**Figure 2:**
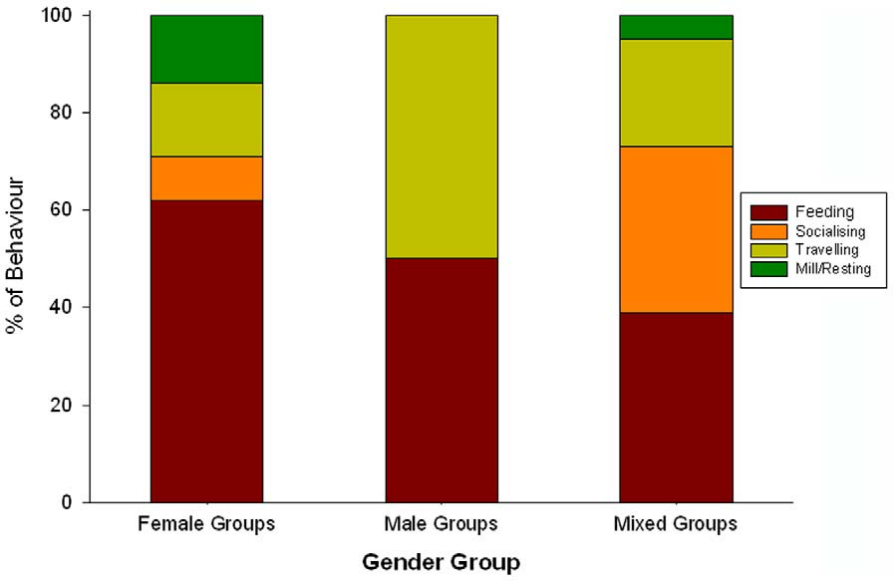
Frequency of dolphin behaviours for female (a), male (b), and mixed-sex (c) groups in the Clarence River estuary. * 76% of the total social interaction time (N = 442) of mixed-sex groups involved aggressive coercive behaviour.

The dolphin gender groups consisted of 1% males, 23% females, 47% mixed-sex, and 29% unknown (*N* = 2382 observations). Male only groups spent equal proportions of time foraging (50%) and travelling (50%) (*N* = 32); females in female groups spent more time foraging (62%) and resting (14%) (*N* = 539) than when in mixed-sex groups (39% and 5% respectively; Figure 2). In mixed-sex groups females spent considerable time interacting with males (34%) (*N* = 1118). Significant differences were observed between the activity budgets of mixed-sex and female groups (X^2^_3_ = 181, df = 3, *P*<0.001) (Figure 2). The mean female group size (excluding singletons) was 3.8 (SE 0.31, range 2–8) while the mean mixed-sex group size was 7.3 (SE 0.56, range 3–34).

### Spatial patterns

**Figure 3:**
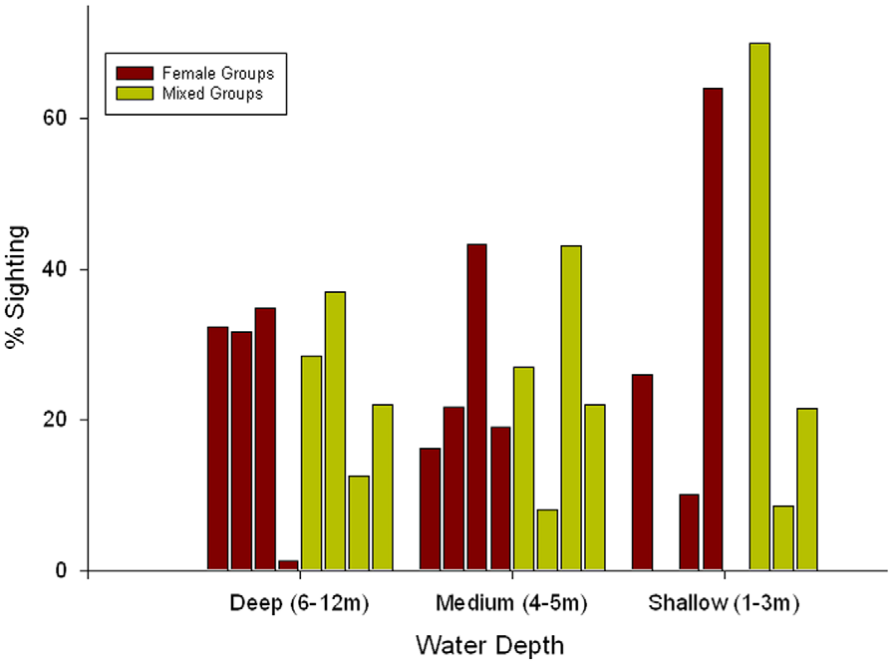
Percentage of dolphin encounters in varying water depths for female and mixed-sex groups in the Clarence River estuary per season. The sum for the four seasons amounts to 100% for each water depth. Seasons are in order Spring, Summer, Autumn and Winter.

Groups of females occurred in all water depth categories, with 30% in deeper water, 34% in medium depths, and 35% in shallow water (X^2^_2_ = 2.2, *P* = 0.35). However, there was a significant difference in the occurrence of mixed-sex groups with respect to depth categories; they were more often found in deeper water (71%) compared with medium (25%) or shallow (4%) water depths (X^2^_2_ = 70.5, df = 2, *P*<0.001). The dolphin gender group sightings per season and water depth were significantly different between female (Kruskal-Wallis *H*_2_ = 91.7, df = 3, P<0.001) and mixed-sex groups (Kruskal-Wallis *H*_2_ = 19.7, df = 3, *P*<0.001) (Figure 3). Female groups occurred in shallow water predominantly in winter, and in deeper water at similar frequencies among the seasons except in winter. They were not observed in shallow water during summer, which is the peak breeding season [42]. Mixed-sex groups were predominantly observed in summer and spring in deeper water.

### Coercive behaviour

In the CR, 76% (*N* = 442) of the total social interaction time of mixed-sex groups involved aggressive coercive behaviour, which represents 14% (*N* = 336) of behaviours in the total behavioural budget recorded for all groups (*N* = 2382). The frequency of male coercive behaviour was significantly affected by water depth, with more occurring in deeper (52%) and medium (48%) depth than in shallow (2%) water (X^2^_2_ = 44.2, df = 2, *P*<0.001).

The tidal phase had a significant effect on the occurrence of coercive behaviour with more coercive behaviour observed at high tide (36%) and at maximum flood (32%), with significantly less coercive behaviour at maximum ebb (24%) and at low (10%) tide (X^2^_3_ = 15.49, df = 3, *P* = 0.001). Significant seasonal differences were evident in the occurrence of coercive behaviour, with more occurring in winter (37%), autumn (32%), and spring (20%) and the least in summer (11%) (X^2^_3_ = 16.56, df = 3, *P* = 0.001). Mean dolphin group size (excluding singletons) during coercive behaviour was not significantly different (Mean 7.2, range 5–11) compared to periods when this behaviour did not occur (Mean = 5.8, range 2–34) (X^2^_2_ = 1.225, *P* = 0.268).

### Habitat use

**Figure 4:**
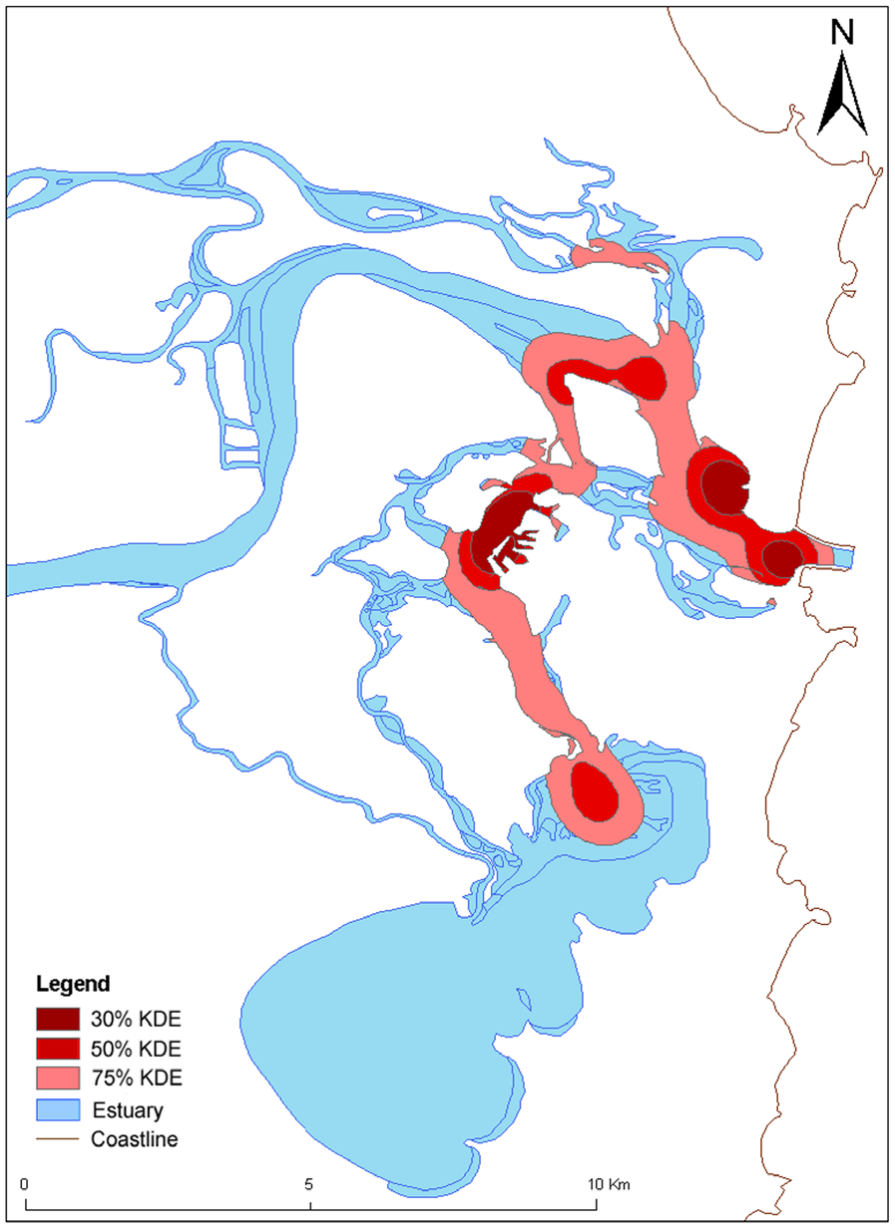
Female groups' kernel density estimator (KDE) for all behaviours from the Clarence River estuary (N = 539).

**Figure 5:**
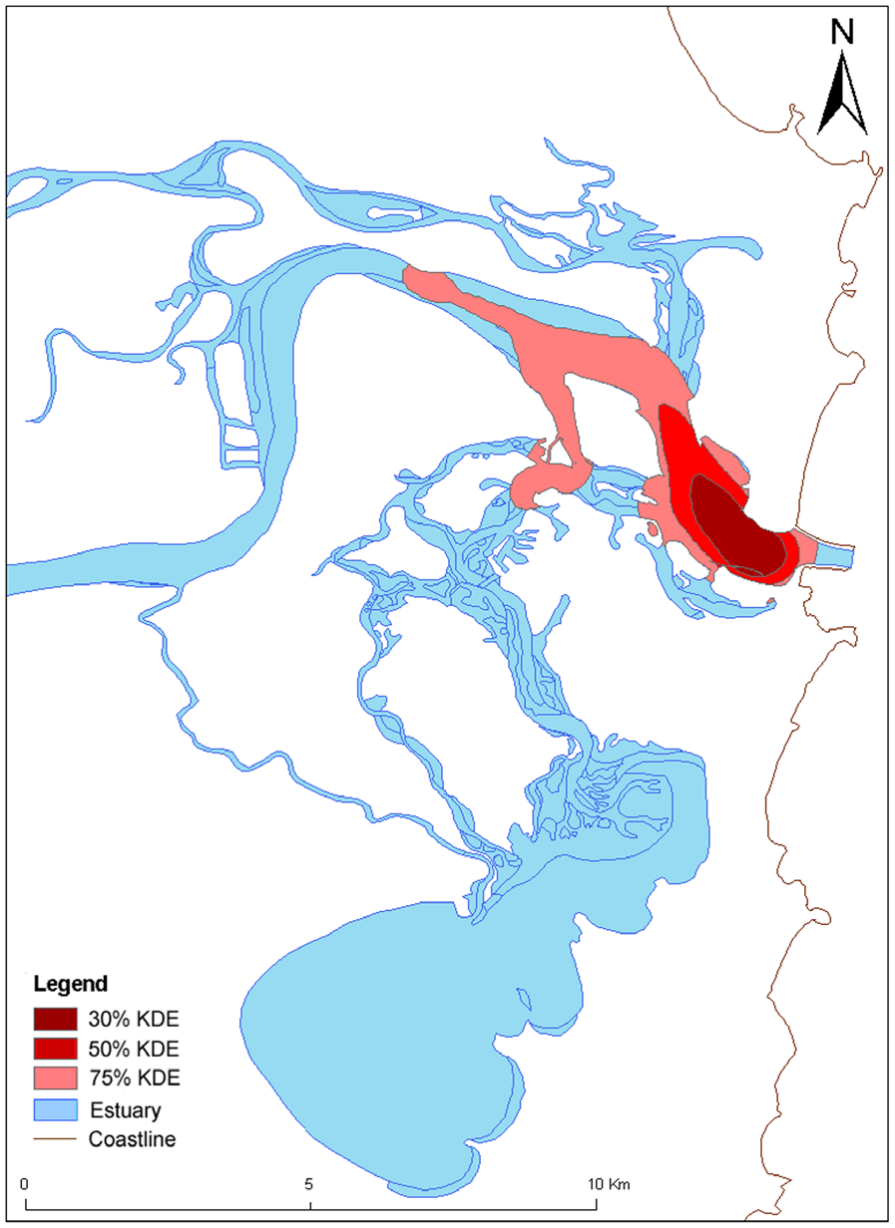
Mixed-sex groups' kernel density estimates (KDE) for all behaviours from the Clarence River estuary (N = 1118).

**Figure 6:**
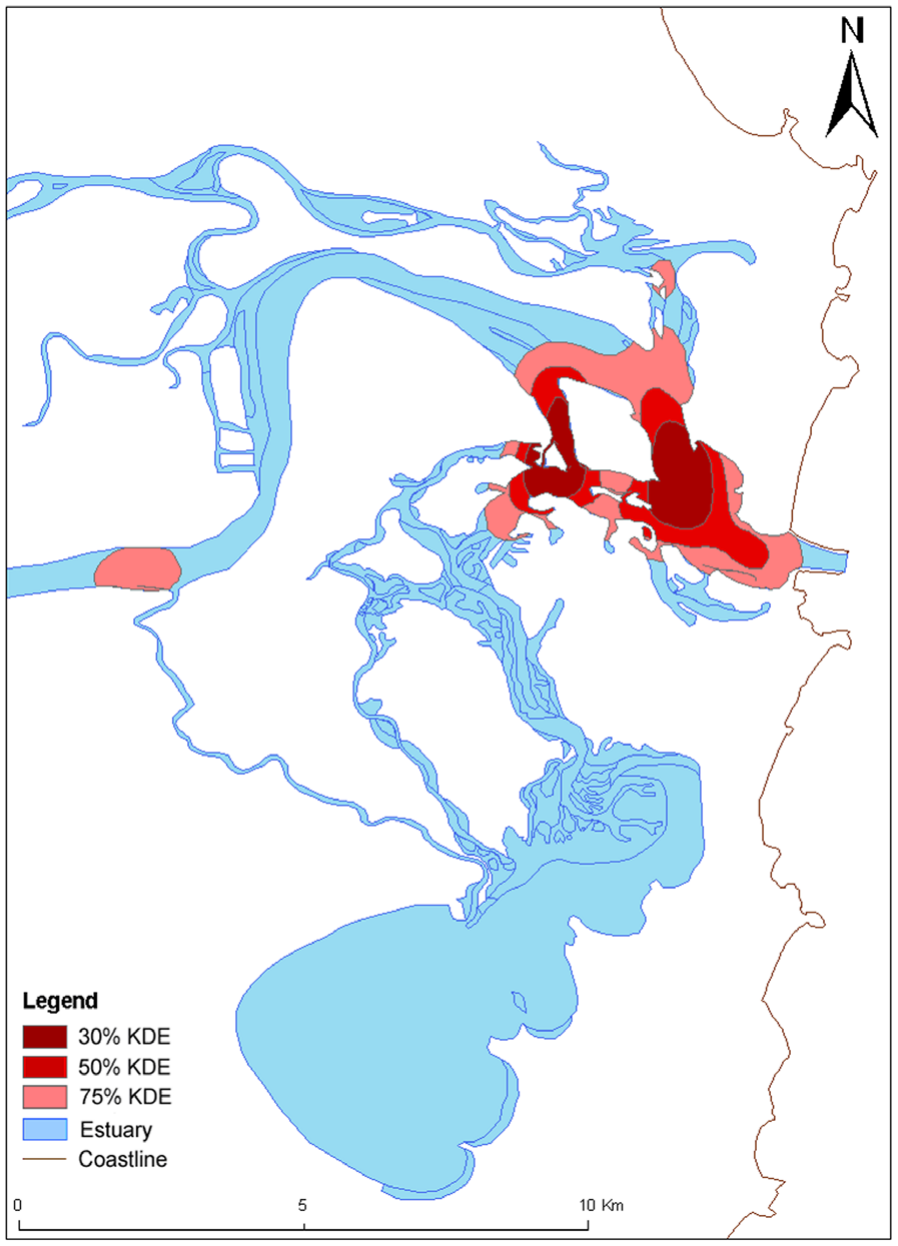
Mixed-sex groups' kernel density estimates (KDE) displaying aggressive coercive behaviour in the Clarence River estuary (N = 336).

Analysis of habitat use using the fixed kernel density estimator analysis for different gender groups revealed that female gender groups (Figure 4) in the CR occupied a larger range of habitats than the mixed-sex groups (Figure 5). Female groups utilized the small, shallow tributaries and travelled for longer distances up these smaller tributaries than the mixed-sex groups, which were concentrated more in the deeper main channel of the estuary (Figure 4). The core use areas for female groups included shallow areas of the channels near sand banks or breakwalls, whereas core use areas for mixed-sex groups were in deeper water in the main estuary channel (Figures 4 and 5). Consequently, coercive behaviour was observed to be concentrated in the main estuary channel in deeper water and in an associated channel in medium water depth (Figure 6).

## Discussion

Female and mixed-sex groups in the Clarence River estuary clearly have different distributions and habitat use patterns leading to social and spatial sexual segregation. Social and habitat segregation has also been found in sperm whales [58], beluga whales (*Delphinapterus leucas*) [59], and botos (*Inia geoffrensis*) [9]. Female bottlenose dolphins groups had a wider distribution and ventured into smaller, shallower channels of the estuary compared to mixed-sex groups, which predominantly used the deeper main estuary channel. Such spatial and habitat segregation and behavioural differences have also been reported in Northern elephant seals (*Mirounga angustirostris*) where females were predominantly foraging in deeper water on pelagic prey whereas males were closer to the coastline foraging mainly on benthic prey, and in Grey seals (*Halichoerus grypus*) [60]. In both of these seal species males are 1.5 to 10 times larger than females [61], whereas bottlenose dolphins do not show a marked sexual dimorphism in size. It is not clear from our study whether behavioural and spatial differences are due to different energy [62] and nutrient requirements due to lactation, or whether coercive behaviour by reproductively active males is contributing to spatial sexual segregation. The coercive behaviour used by male dolphins in our study is of particular interest, and its effect on the distribution of the female dolphin groups is discussed below, with respect to water depth and its relevance for mother and calf groups.

### Sexual segregation

#### Grouping patterns and activity budgets

Groups of female *T. aduncus* have been found to associate more with other females in a similar reproductive state than with females in different reproductive states [63]. This was also observed in the CR, with females with calves tending to associate in a group with other females with calves (C. Fury, pers. obs.). Mixed-sex groups were found all year round, however, they were predominately found in the peak-breeding season of spring and summer (*N* = 19 and 17 respectively). CR female dolphins in female groups spent a greater proportion of their time foraging (CR: 62%), compared to when they were in a mixed-sex group (CR: 39%) (Figure 2). A study on humpback whales (*Megaptera novaeangliae*) found that when female and calf pairs associated with multiple males they increased their time spent travelling and decreased their time at rest [64], which would be energetically costly to both the mother and her calf. Hence, female dolphins might segregate from males because of different activity budgets.

Differences in time spent feeding could however also be a reflection of higher food selectivity of females, less food availability in areas frequented by females and/or higher energetic demands during the period of offspring dependence. We are unable to identify the main causes of this behavioural difference. However, it has been proposed that sexual differences in activity budgets could cause social segregation [29], [30]. To investigate whether sexual differences are large enough to lead to segregation, males in all male groups and mixed-sex groups should be compared to females in nursery groups and mixed-sex groups.

#### Predation risk

Females with calves are likely to be more vulnerable to predation and should therefore use areas that are relatively safe [65]. In Shark Bay, Western Australia, for example, tiger sharks (*Galeocerdo cuvier*) that are preying on calves, are found mostly in shallow water and their density is much greater in the warmer months (September–May) than during the cooler months (June–August) [66]. Studies on bottlenose dolphins and the effect of food availability and tiger shark predation [66], [67] found that when shark abundance was high, foraging dolphins greatly reduced their use of dangerous, but productive, shallow water patches and were found in relatively safer deep water areas. However the Shark Bay female dolphins' reproductive success was higher if they frequented the shallow water areas [68]. This suggests that the distributions of foraging dolphins may reflect a trade-off between predation risk and food availability.

In the CR, the most common potential predators of dolphins would be bull sharks that are known to occur in estuaries [69], [70] [71], but most bull sharks that frequent estuaries are juveniles [14], [69], [72]. In the CR, the female dolphins did not avoid any particular water depth and all of the estuary is <20 m depth. Given that we do not know the distribution of adult bull sharks and their foraging patterns we do not now whether females with calves frequent estuaries to avoid attacks on their calves by adult bull sharks.

#### Prey selection

The preferred prey items of CR dolphins are sea mullet (*Mugil cephalus*) and sand whiting (*S. ciliata*) [35]. Larger numbers of fish have been reported in shallow water compared to deep water in Australian estuaries. Specifically, a study in the Clarence River showed that the shallow vegetated habitats in the marine region have the greatest diversity and highest abundances of fish in the estuary [73], therefore creating potentially more effective foraging habitats for mothers and calves. Female groups often frequented the Oyster Channel, a shallow tributary in the CR estuary where no mixed-sex groups were observed during surveys. Fish surveys in that channel have shown that the mean fish abundance peaks are in winter and spring [31], when females, often with calves, were observed in the channel during surveys. Therefore, the use of these shallow water areas in the estuary by mothers and calves could be directly linked to the availability of prey species and may not be primarily driven by predator avoidance. In a monomorphic species, like *T. aduncus*, lactating females will have higher metabolic needs and might thus be expected to spend different proportions of their time feeding or select different prey items compared to males or non-reproducting females [1], [74]. Furthermore, calves need to learn to catch prey through their associations with mothers. Catching smaller prey, such as sand whiting (*Sillago ciliata*), is likely to be easier for calves in shallow compared to deep water. Calves begin to chase fish after a few months of age [75], [76]. If fish are easier to catch and plentiful the question remains why male or mixed-sex groups do not seem to frequent these areas much. Breed *et al.* [60] found marked sexual segregation in grey seals and suggested that males and females might avoid scramble competition for food during important times of the year. Further investigation into diet preferences, scramble competition, and optimal foraging strategies by the different adult classes of dolphins is needed.

#### Male Avoidance

Male harrassment of females for breeding has been shown in various species, including but not limited to the boto (*I. geoffrensis*) [9], dusky dolphins (*Lagenorhynchus obscurus*) [77], Hector's Dolphin (*Cephalorhynchus hectori*) [6], grizzly bears [78], polar bears [79], and ungulates [11], [13]. Male aggressive behaviour can drive females to alter their habitat use of an area, as was shown in a study on wild Trinidadian guppies (*Poecilia reticulata*) [80]. Male harassment and forced copulations are considered costly for females [81] as they and their calves can be injured or killed by males [9], [81]–[84]. Therefore, female bottlenose dolphins in the CR may be trying to avoid injury to themselves and their calves, especially during aggressive coercive behaviour, and thus segregate into shallow waters that are not frequented by these males.

Most coercive behaviour recorded in the CR estuary occurred at high tide or flood tide in deeper water or medium depth water, while mixed-sex groups only occurred during low tide or ebb tide in deeper water. In shallower waters, the males might not be able to manoeuvre to mate effectively. The only times (N = 5) that herded females were observed escaping from the males was into shallower areas (C. Fury, unpublished obs.). Female dusky dolphins also have a lower likelihood of being harassed by males when occupying shallow waters and therefore shallow waters were considered to be a refuge from males [77].

Male coercive encounters in the CR often lasted for hours (range 18–159 mins, mean 68 mins). Our study showed that in winter (non-breeding season), when more coercive behaviour occurred, females and calves preferred shallow water, which may have been to avoid males. In contrast, in the summer peak-breeding season females were not observed in shallow water and occurred more often in the deeper water in mixed-sex groups, possibly to aid in mating and conception. Hence, females might segregate from males to avoid potential costs of associating with males in mixed-sex groups, while with a dependent calf.

## Conclusions

Our study suggests that the bottlenose dolphins in the CR estuary are socially and spatially segregated. There are several factors that potentially contribute to the sexual segregation found in this species, including differences in activity budgets between males and females, the need for calves to learn to forage and capture prey in shallow waters, females with calves avoiding predation, and female avoidance of harrassment by males. Given the marked differences in activity budgets between female only and mixed-sex groups, it could be costly for females to frequent mixed-sex groups, except for reproduction. Habitat use by female dolphin groups in the CR estuary suggests that shallow areas of the estuary may provide a sanctuary from aggressive males, and access to suitable prey items and prey density for mothers and their calves, or a combination of these factors.
